# Hair disorders associated with post-COVID-19 infection in females: a cross-sectional study

**DOI:** 10.1007/s11845-023-03509-0

**Published:** 2023-09-14

**Authors:** Nehal El Hendawy Ali Awad, Zakaria M. Obaid, Mohamed S. Zaky, Mohamed L. Elsaie

**Affiliations:** 1 Department of Dermatology, Venereology and Andrology, Damietta Faculty of Medicine, Al-Azhar University, Damietta, Egypt; 2 Department of Dermatology, Venereology and Andrology, Medical Research and Clinical Studies Institute, National Research Centre, Giza, Egypt

**Keywords:** Alopecia, Telogen effluvium, Trichoscopy

## Abstract

**Background:**

Coronavirus disease (COVID-19) currently named SARS-CoV-2 is a contagious disease caused by a coronavirus. The virus may infect the hair follicles directly or indirectly through systemic changes in the immune or hormonal systems.

**Aims:**

In the current study we aimed to determine the prevalence of hair disorders in females infected with COVID-19.

**Methods:**

Data was collected using a questionnaire covering four main domains: personal data, past medical history, COVID-19 history and treatment, and existence of any hair problems and their management. No identifier or sensitive data were collected. Those complaining of hair loss were subjected to complete general and local hair examination using trichoscopy to confirm hair loss.

**Results:**

Hair problems were reported in 307 (61.4%) of COVID-19-infected female subjects. A total of 68.1% patients reported that hair loss existed and increased after COVID-19; 29.6% reported their hair problems only post-COVID-19 while 2.3% had hair shedding issues during infection only. The main reported hair problems were telogen effluvium (60.8%), increased gray hair (13.8%), seborrheic dermatitis (5.6%) trichotillomania (3.6%), and alopecia areata (2.2%).

**Conclusion:**

In conclusion, we reported prevalence of post-COVID hair fall that was confirmed by trichoscopy and which affected approximately 61.4% of infected females.

## Background

The coronavirus disease 2019 (COVID-19) has become the most emergent health issue globally. SARS-CoV-2, the pathogen of COVID-19, directly infects multiple tissues and organs, possibly involving skin and hair follicles, and profoundly affects the immune system [1, 2].

Telogen effluvium (TE) and hair loss following bacterial, viral, or protozoal infection had been earlier reported and was of major concern during the 1918 influenza epidemic [3]. Literature review in terms of the impact of COVID-19 infection on the hair follicle reveals hair loss caused during and post-recovery, majorly manifesting as TE [1–3].

The virus may infect the hair follicles directly or indirectly through systemic changes in the immune or hormonal systems [4]. Furthermore, it is plausible to believe that alopecia may influence or predict the severity and course of COVID-19. A growing number of reports have focused on the clinical manifestations and pathophysiology of COVID-19-associated hair diseases [4, 5].

In the current study we aimed to determine the prevalence of hair disorders in females infected with COVID-19. For that we enrolled 500 female patients post-COVID-19 and they were asked questions about pre- and post-pandemic hair diseases. All patients were subjected to online questionnaire containing 25 questions which have been divided into four domains (personal data, medical past history, diagnoses and treatment of COVID-19 infections, hair problems and their management).

## Methods

This was a multi-center, cross-sectional study conducted from January 2022 to January of 2023. A total of 500 female participants attending dermatology clinics at university as well as ministry of health hospitals were included in the study after their consenting. The study followed the Helsinki declaration principals. Ethical approval was obtained from the institutional review board of Damietta Faculty of Medicine (Al-Azhar University). Written informed consent was obtained from every patient at recruitment. Inclusions included females aged 18–55 years of age whom were diagnosed with COVID-19 infection confirmed by using the reverse transcription polymerase chain reaction (RT-PCR) test of pharyngeal and nasal swabs (only those who finished the management protocol and had been discharged at least 6 months prior to joining the study). Those who were aged less than 18 years or above 55 years were excluded.

Data was collected using a questionnaire covering four main domains: personal data, past medical history, COVID-19 history and treatment, and existence of any hair problems and their management. No identifier or sensitive data were collected. Those complaining of hair loss were subjected to complete general and local hair examination using trichoscopy to confirm hair loss. Dermoscopic evaluation (DermLite DL4) and imaging were reproduced and saved.

The study was conducted in accordance with the Declaration of Helsinki and upon approval by the Local Ethical Committee—Al Azhar University IRB (00012367–21-06–005). Statistical analysis using continuous variables were presented as mean ± SD (standard deviation) for parametric data and median (min–max) for non-parametric data. Qualitative data were described using number and percent. Association between categorical variables was tested using Chi-square test. For all statistical tests *p*-value > 0.05 was considered not-significant and *p*-value < 0.05 was considered significant. All data were analyzed using IBM SPSS Statistics for Windows, Version 26, and Microsoft Excel 365.

## Results

A total of 500 patients were enrolled in the study using a data collection sheet. Patients’ ages ranged from 18 to 55 years with a mean age of 37.5 ± 8.4 years. A total of 360 (72%) patients were married and 114 (22.8%) patients were single. Smoking was reported among 16 (3.2%) patients. A total of 232 (46.4%) patients were health care workers (HCWs). Regarding chronic diseases, 15 (3%) patients were diabetic, 55 (11%) patients had hypertension, 20 (4%) had a hypercoagulable condition, while 384 (76.8%) patients had no chronic health problem. Only 9 (1.8%) of patients had a psychiatric health condition and 17 (3.4%) complained of hypothyroidism (Table 1).

**Table 1:** Sociodemographic characteristics of the studied cases

**Sociodemographic characteristics**	***n* = 500**	**%**
**Age/years** ** 19–30** ** 31–40** ** 41–55**	138 222 140	27.6 44.4 28.0
**Occupation** ** Not working** ** Retired** ** Health care worker** ** Governmental employee** ** Special business** ** Others**	92 9 232 132 19 16	18.4 1.8 46.4 26.4 3.8 3.2
**Marital status** ** Single** ** Married** ** Divorced** ** Widow**	114 360 10 16	22.8 72.0 2.0 3.2
**Educational level** ** Middle or lower** ** Secondary** ** Higher education**	26 32 442	5.2 6.4 88.4
**Income** ** Low** ** Middle** ** High**	6 448 46	1.2 89.6 9.2
**Special habits** ** Smoking**	16	3.2

The COVID-19 infection data among study patients demonstrated that among the 500 patients studied, 78 (15.6%) were hospitalized, and 10 (2%) needed ICU admission. Medications used during COVID-19 infection included vitamins (89%), antipyretics (85.8%), antibiotics (76.6%), expectorants (43.2%), and anticoagulants (37.4%).

All participants (100%) were diagnosed mainly by PCR, 34% diagnosed further clinically, and 23% needed CT scan for diagnosis confirmation. The five main symptoms found among participants were bone and muscle aches (85.4%), fever (74.2%), loss of smell and taste (714%), cough (62.0%), and shortness of breath (51.8%) (Table 2).

**Table 2:** COVID-19 diagnosis and symptoms among studied cases

**Method of diagnosis**	***n* = 500**	**%**
**Clinically** ** PCR** ** CT scan** ** Others**	170 500 119 16	34.0 100.0 23.8 3.2
**Symptoms during COVID-19 infection**		
** Fever** ** Bone and muscle aches** ** Cough** ** Shortness of breath** ** Eye inflammation** ** Stomach aches** ** Diarrhea** ** Vomiting** ** Severe respiratory symptoms** ** Loss of smell and taste** ** Others**	371 427 310 259 71 142 155 64 73 357 68	74.2 85.4 62.0 51.8 14.2 28.4 31.0 12.8 14.6 71.4 13.6
**Symptoms needed hospitalization**	78	15.6
**Symptoms needed ICU admission**	10	2.0

*COVID-19* coronavirus disease, *PCR* polymerase chain reaction, *CT* computerized tomography

**Fig. 1:**
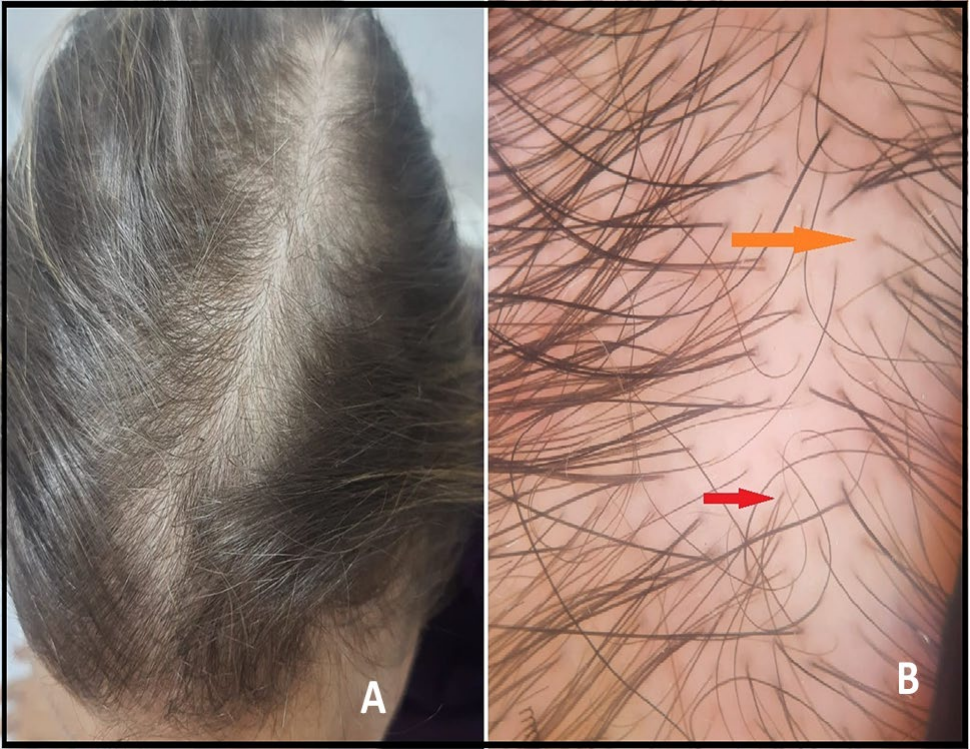
**A** Clinical image of a 32-year-old female showing reduced hair density with widened hair over the vertex and mid-frontal scalp. **B** Dermoscopy image showing great variability in the thickness of hair shaft or hair shaft diversity, vellus hair (red arrow), and peripilar sign (orange arrow) suggestive of androgenetic alopecia

**Fig. 2:**
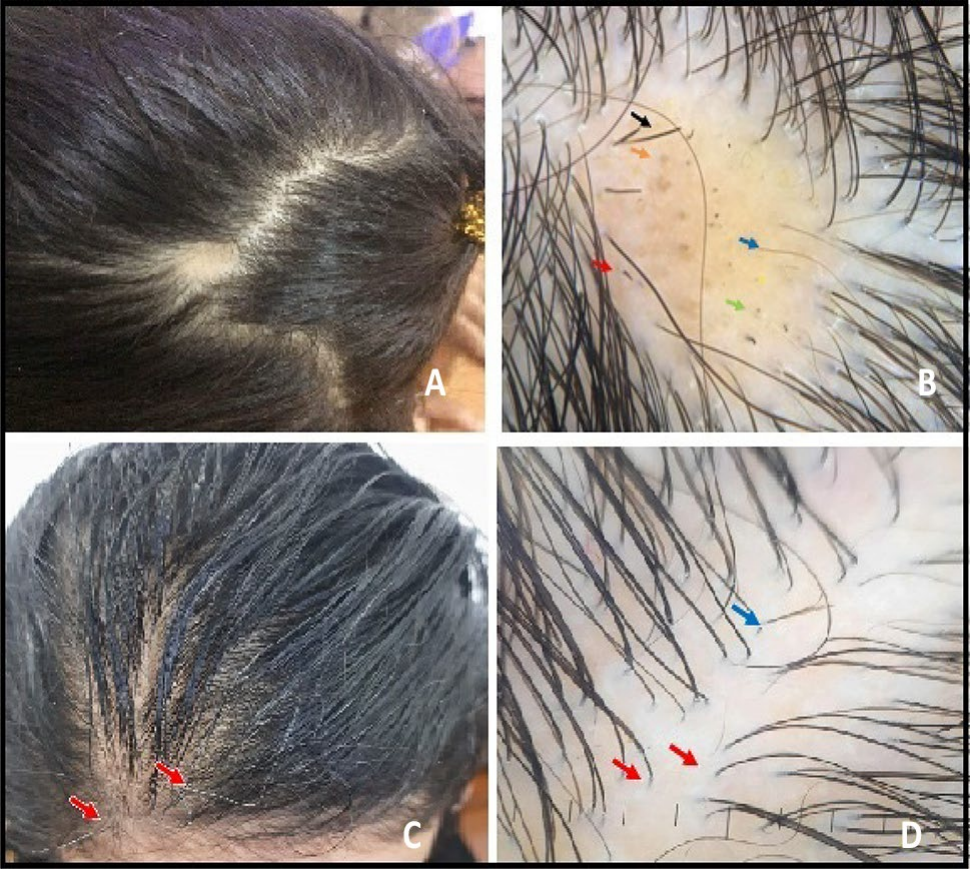
**A** Clinical image showing localized shedding of hair in the frontal area of the scalp surrounded by preserved hairs in a 22-year-old female. **B** Dermoscopy image showing yellow dots (orange arrow), black dots (green arrow), broken hair (red arrow), and tapering hair (black arrow) suggestive of alopecia areata. **C** Clinical image of a 21-year-old female showing reduced hair density all over the scalp with gray hair appear recently (red arrows). **D** Dermoscopy image showing upright re-growing hair (blue arrow), single hair emerging from the hair follicles (red arrows), and no hair shaft diversity suggestive of telogen effluvium

**Fig. 3:**
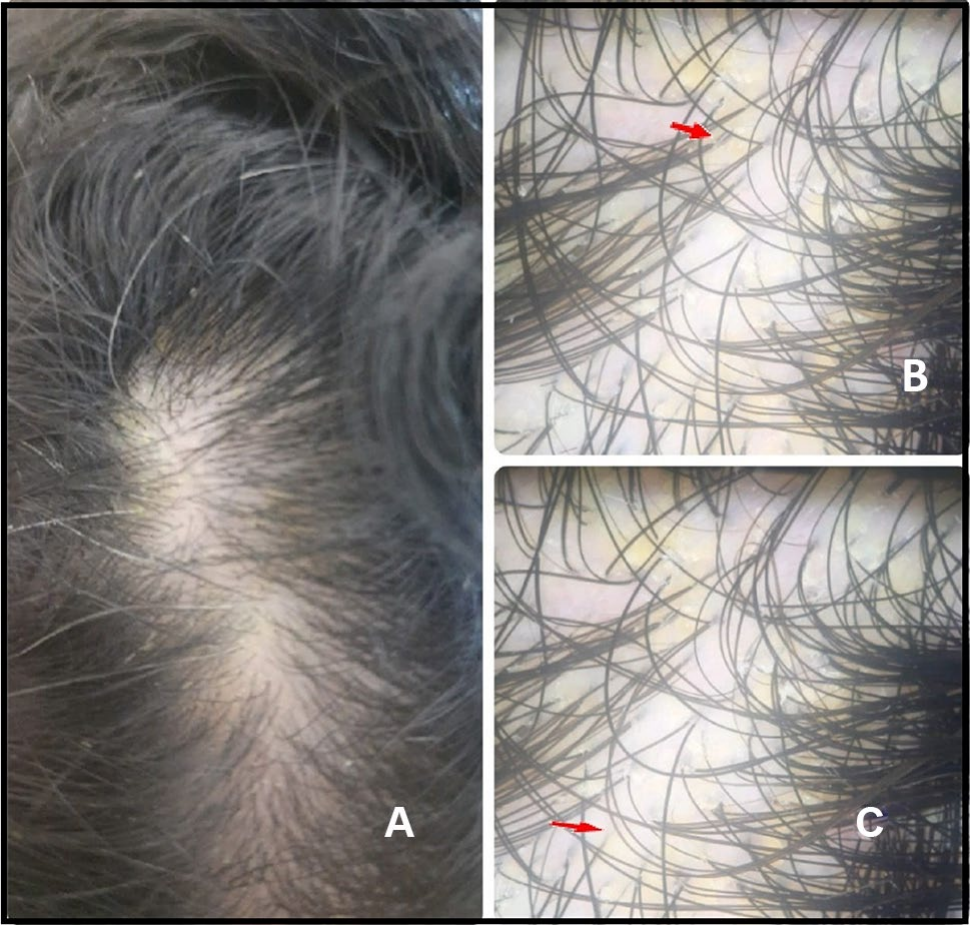
**A** A 21-year-old female patient complaining of clinical image showing diffuse scales of frontal and middle areas of the scalp with preservation of hair structure and density. **B** and **C** Dermoscopy images showing yellowish scales among follicular units (red arrow) suggestive of seborrheic dermatitis

Hair problems were reported in 307 (61.4%) of COVID-19-infected female subjects. A total of 68.1% patients reported that hair loss existed and increased after COVID-19; 29.6% reported their hair problems only post-COVID-19, while 2.3% had hair shedding issues during infection only. The main reported hair problems were telogen effluvium (60.8%), increased gray hair (13.8%), seborrheic dermatitis (5.6%), trichotillomania (3.6%), and alopecia areata (2.2%). Hair loss occurred after 2–3 months of COVID-19 infection among 93 (18.6%) cases, while 213 (42.6%) patients noticed hair loss after 6 months of the infection. Of the participants with hair problems, 65.2% did not use any treatment; 8.2% of them used treatment based on past experiences; 7.6% received prescription medications through physician telemedicine calls; and only 10.4% visited a dermatologist (Figs. 1, 2, 3 and Table 3).

**Table 3:** Hair loss data among studied cases

**Hair loss data**	***n* = 500**	**%**
**Complaining of hair problems before and or/after**	307	61.4
** Yes before and become worse after infection** ** Yes post-COVID-19 only** ** Yes, only during infection only**	209 91 7	68.1 29.6 2.3
**Hair problems** ** Telogen effluvium** ** Alopecia areata** ** Seborrheic dermatitis** ** Increased gray hair** ** Trichitollamania**	304 11 28 69 18 164	60.8 2.2 5.6 13.8 3.6 32.8
**No treatment used** ** Used treatment based on past experience** ** Recommended treatment by others** ** Based on physician telephone call** ** Visit to non-specialist doctor** ** Visit to a dermatologist**	326 41 7 38 36 52	65.2 8.2 1.4 7.6 7.2 10.4

*COVID-19* coronavirus disease

The prevalence of hair loss was significantly higher among those in the 31–40-year age group when compared to other age groups (*p* < 0.001). In addition, hair loss was significantly higher among HCWs, divorced, and widow females (Table 4). The prevalence of post-infection hair loss was significantly higher among females complaining of a hypercoagulable state while a significantly less prevalence of hair loss was found among females with hypertension and those complaining of a psychiatric condition (*p* < 0.01). Other factors were insignificantly associated with post-infection hair loss. More prevalent hair loss was reported among those who needed hospital and ICU admission as well as among those complaining of severe respiratory symptoms and shortness of breath (*p* = 0.001, *p* = 0.002, respectively). Of notice hair loss was significantly higher among those who used antibiotics for COVID treatment (*p* = 0.027) (Tables 5, 6 and 7) .

**Table 4:** Comparison of sociodemographic characteristics among studied cases

**Sociodemographic characteristics**	**No hair problems** ***n* = 193 (%)**	**Hair problems** ***n* = 307 (%)**	**Test of significance**
**Age/years** ** 19–30** ** 31–40** ** 41–55**	67 (34.7) 52 (26.9) 74 (38.3)	71 (23.1) 170 (55.4) 66 (21.5)	*χ*^2^ = 39.35 *p* < 0.001*
**Occupation** ** Not working** ** Retired** ** Health care worker** ** Governmental employee** ** Special business**	56 (29.0) 9 (4.7) 46 (23.8) 63 (32.6) 9 (4.7) 10 (5.2)	36 (11.7) 0 186 (60.6) 69 (22.5) 10 (3.3) 6 (2.0)	*χ*^2MC^ = 77.18 *p* < 0.001*
**Marital status** ** Single** ** Married** ** Divorced** ** Widow**	54 (28.0) 139 (72.0) 0 0	60 (19.5) 221 (72.0) 10 (3.3) 16 (5.2)	*χ*^2MC^ = 77.18 *p* < 0.001*
**Educational level** ** Middle or lower** ** Secondary** ** Higher education**	13 (6.7) 32 (16.6) 148 (76.7)	13 (4.2) 0 294 (95.8)	*χ*^2^ = 57.21 *p* < 0.001*
**Income** ** Low** ** Middle** ** High**	6 (3.1) 187 (96.9) 0	0 261 (85.0) 46 (15.0)	*χ*^2MC^ = 40.33 *p* < 0.001*
**Special habits** ** Smoking**	16 (8.3)	0	*χ*^2^ = 26.29 *p* < 0.001*

*χ*^*2*^: Chi-square test

^*^Statistically significant

**Table 5:** Comparison of associated comorbidities among studied cases

**Associated comorbidities**	**No hair problems** ***n* = 193 (%)**	**Hair problems** ***n* = 307 (%)**	**Test of significance**
**Hypertension** **Diabetes mellitus** **Hypercoagulation** **Psychiatric conditions** **Hypothyroidism**	**37 (19.2)** **9 (4.7)** **0** **9 (4.7)** **7 (3.6)**	**18 (5.9)** **6 (2.0)** **20 (6.5)** **0** **10 (3.3)**	***χ***^**2**^ = 21.44, *p* < 0.001* ***χ***^**2**^** = 2.98, *p* = 0.084** ***χ***^**2**^ = 13.09, *p* < 0.001* ***χ***^**2**^ = 14.58, *p* < 0.001* ***χ***^**2**^** = 0.049, *p* = 0.806**

*χ*^*2*^: Chi-square test

^*^Statistically significant

**Table 6:** Comparison of COVID-19 symptoms among studied cases

**Symptoms during COVID-19 infection**	**No hair problems** ***n* = 193 (%)**	**Hair problems** ***n* = 307 (%)**	**Test of significance**
**Fever** **Bone and muscle aches** **Cough** **Shortness of breath** **Eye inflammation** **Stomach aches** **Diarrhea** **Vomiting** **Severe respiratory symptoms** **Loss of smell and taste**	148 (76.7) 175 (90.7) 124 (64.2) 117 (60.6) 26 (13.5) 61 (31.6) 78 (40.4) 44 (22.8) 25 (8.1) 123 (63.7)	223 (72.6) 252 (82.1) 186 (60.6) 142 (46.3) 45 (14.7) 81 (26.4) 77 (25.1) 20 (6.5) 48 (24.9) 234 (76.2)	*χ*^2^ = 1.013, *p* = 0.314 *χ*^2^ = 7.01, *p* = 0.008* *χ*^2^ = 0.675, *p* = 0.411 *χ*^2^ = 9.79, *p* = 0.002* *χ*^2^ = 0.137, *p* = 0.711 *χ*^2^ = 1.58, *p* = 0.207 *χ*^2^ = 13.03, *p* < 0.001* *χ*^2^ = 28.15, *p* < 0.001* *χ*^2^ = 26.59, *p* < 0.001* *χ*^2^ = 9.05, *p* = 0.003*
**Symptoms needed hospitalization**	18 (9.3)	60 (19.5)	*χ*^2^ = 10.71, *p* = 0.001*
**Symptoms needed ICU admission**	0 (0.0)	10 (3.3)	*χ*^2^ = 6.42, *p* = 0.001*

*χ*^*2*^: Chi-square test, *ICU* intensive care unit

^*^Statistically significant

**Table 7:** Comparison of laboratory findings and medications used during COVID-19 among studied cases

**Laboratory findings during COVID-19 infection**	**No hair problems** ***n* = 193 (%)**	**Hair problems** ***n* = 307 (%)**	**Test of significance**
CBC CRP ESR LDH D-dimer Serum ferritin	84 (43.5) 35 (18.1) 25 (13.0) 0 27 (14.0) 17 (8.8)	174 (56.7) 90 (29.3) 15 (4.9) 10 (3.3) 56 (18.2) 18 (5.9)	*χ*^2^ = 8.21, *p* = 0.004* *χ*^2^ = 7.90, *p* = 0.005* *χ*^2^ = 10.48, *p* = 0.001* *χ*^2^ = 6.42, *p* = 0.01* *χ*^2^ = 1.55, *p* = 0.214 *χ*^2^ = 1.58, *p* = 0.209
**Medications during COVID-19 infection**	**No hair problems** ***n* = 193 (%)**	**Hair problems** ***n* = 307 (%)**	**Test of significance**
Antibiotics Antipyretics Vitamins Expectorant Antitussive Corticosteroids Anticoagulant	158 (81.9) 159 (82.4) 161 (83.4) 60 (31.1) 34 (17.6) 66 (34.2) 65 (33.7)	225 (73.3) 270 (87.9) 284 (92.5) 156 (50.8) 143 (46.6) 97 (31.6) 122 (39.7)	*χ*^2^ = 4.86, *p* = 0.027* *χ*^2^ = 3.01, *p* = 0.083 *χ*^2^ = 9.99, *p* = 0.002* *χ*^2^ = 18.79, *p* < 0.001* *χ*^2^ = 43.47, *p* < 0.001* *χ*^2^ = 0.365, *p* = 0.546 *χ*^2^ = 1.86, *p* = 0.173

*χ*^*2*^: Chi-square test, *CBC* complete blood picture, *CRP* C reactive protein, *ESR* erythrocyte sedimentation rate, *LDH* lactate dehydrogenases

^*^Statistically significant

## Discussion

The current study was conducted to assess the prevalence of hair loss after COVID-19 infection and the factors associated with post-COVID-19 hair loss among 500 female study patients. In the present study, only confirmed COVID-19 cases were analyzed. Hair problems were reported in more than half of the participants (307; 61.4%) whom 68.1% of them reported worsening of their hair loss following contracting COVID; 29.6% of them reported noticing hair problem post-COVID only while 2.3% developed transient hair loss only during the infection.

Results in the current study were in accordance with a major Saudi study that assessed the prevalence of hair loss following COVID-19 infection as well as the factors associated with post-COVID-19 which revealed that nearly half of their participants (48.5%) noticed an increase in hair loss of more than 120 hairs per day after COVID-19 infection, and half of the participants (52.6%) reported hair accumulation on a pillow [6]. Another study recruiting a total of 806 participants reported that 52.7% experienced hair loss after COVID-19 infection. Age, gender, high temperature during, and the presence of hair loss prior to infection were significantly associated with the incidence of telogen effluvium (TE) [7]. According to a study conducted in Brazil via remote SMS messages and electronic form fill ups by patients of COVID-19 saved on the national registry, hair loss was the most frequently reported post-COVID-19 manifestation in 2800 subjects that comprised 48% of the studied cohort [8].

A meta-analysis performed on 15 published studies included 47,910 patients (age 17–87 years). The included studies defined long-COVID as ranging from 14 to 110 days post-viral infection. It was estimated that 80% of the infected patients with SARS-CoV-2 developed one or more long-term symptoms. The five most common symptoms were fatigue (58%), headache (44%), attention disorder (27%), hair loss (25%), and dyspnea (24%) [9].

The main reported hair problems in the current study were telogen effluvium (TE) (60.8%), increased gray hair (13%), seborrheic dermatitis (SE) (5.6%), trichotillomania (3.6%), and alopecia areata (AA) (2.2%). There is a wide discrepancy between studies regarding the prevalence of different related hair problems in post-COVID-19. An online-based self-administered cross-sectional study on 404 medical students in Bangladesh found prevalence of TE, AA, and SD to be 61, 25, and 58%, respectively [10]. In Turkey, one study reported the prevalence of TE, AA, and SD to be 28, 2.8, and 20%, respectively [11], while another conducted on 2171 post-COVID-19 patients found that TE (85%) was the most common type of hair loss followed by worsening of androgenetic alopecia (AGA) (7%) [12]. A total of 321 (29.13%) patients developed graying/whitening of hair after getting infected with COVID-19.

TE is a type of reactive non-scarring hair loss that causes hair shedding and is more common in women. TE is a harmless disorder that has been linked to hormonal changes, extreme dieting, infections, and the use of anticoagulant medications, prescribed for treating COVID-19 patients [13].

In the current study, there was higher significance of hair loss among users of vitamins, expectorants, and antitussive medications (*p* < 0.05) while a non-significant prevalence of hair loss was encountered among users of anticoagulants (*p* = 0.173). Of notice hair loss was significantly higher among those who used antibiotics for COVID treatment (*p* = 0.027). Some medications used to treat COVID infection were reported to trigger telogen effluvium (enoxaparin, hydroxychloroquine, azithromycin, etc.). On the other hand, there is currently conflicting evidence regarding their mechanisms. Regarding the prophylactic anticoagulant therapy used in patients affected by SARS-CoV-2, most studies reported important associations of hair loss in these patients, possibly due to collagen degeneration of the follicular sheath [14].

The COVID-19 pandemic has increased the levels of anxiety and stress-related disorders among individuals which contributed to hair shedding and TE. Rates of acute hair shedding during the pandemic increased for which patients requested treatments to break such cycle. Interestingly, we found that only 25.2% of patients sought medical advice through phone calls and visit to general physician or dermatologists, similar to the rate of 18.9% reported by Turkmen et al. [11] and 22.9% reported by Alsalhi et al. [15].

In the current report, more prevalent hair loss was reported among those who needed hospital and ICU admission as well as among those complaining of severe respiratory symptoms and shortness of breath (*p* = 0.001, *p* = 0.002, respectively). Regarding the severity of the infectious episode, different studies revealed a positive correlation of hair loss and severity of infection requiring hospital or ICU admission and related this to increase in proinflammatory cytokines [14].

In specialized studies, smoking was frequently associated with the appearance of TE, due to influencing follicular apoptosis due to altering the sensitivity of acetyl choline receptor [10]. In our case smoking did not appear to affect hair loss. The relationship between smoking and COVID-19 seems to be very controversial while smokers are susceptible to COVID-19; some reports suggested that it may also have a protective effect on the respiratory tract [16, 17].

A number of mechanisms had been proposed for post-COVID hair loss involving direct endothelial cell damage; in addition, aggravation of existing hair loss was related to SARS-CoV-2 virus acting on the transmembrane serine protease 2 genes (TMPRSS2)—which has a role in the regulation of androgen pathways [4]. Another cause of hair loss related the severity of the viral inflammation and cytokine storm to hair matrix cell destruction [5]. Moreover, perifollicular inflammation manifested by the accumulation of activated macrophages and mast cell degranulation in the context of psychological stress as well as the ischemia and vascular decompensation caused by the hypercoagulability state and microthrombus formation were described as major culprits in inducing hair loss [18]. Moreover, a recent study reported an increased incidence of hair shedding following COVID-19 vaccinations [19].

The following study is limited by its cross-sectional nature and by its gender bias on a female population in a localized community. Objective measures such as inflammatory biomarkers and oxygen saturation which are detrimental for the severity of infection have not been measured. Moreover, a lack of scalp biopsy remains to be a limitation.

## Conclusion

In conclusion, we reported prevalence of post-COVID hair fall that was confirmed by trichoscopy and which affected approximately 61.4% of infected females. Other factors, such as stress and infection, cannot be excluded and remain to be further investigated by larger multicenter studies.

## Data Availability

The data that support the findings of this study are available from the corresponding author upon reasonable request.

## Abbreviations

COVID-19: Coronavirus disease (COVID-19)
TE: Telogen effluvium
AA: Alopecia areata
NK cells: Natural killer cells
IFN: Interferon
HCW: Health care workers
TMPRSS2: Transmembrane protease, serine 2
CK: Creatine kinase
CRP: C-reactive protein
DM: Diabetes mellitus
